# Linear and Non-Linear Visual Feature Learning in Rat and Humans

**DOI:** 10.3389/fnbeh.2016.00235

**Published:** 2016-12-23

**Authors:** Christophe Bossens, Hans P. Op de Beeck

**Affiliations:** Laboratory for Biological Psychology, KU LeuvenLeuven, Belgium

**Keywords:** behavior, object discrimination, feature learning, exclusive-or relation, learning curves

## Abstract

The visual system processes visual input in a hierarchical manner in order to extract relevant features that can be used in tasks such as invariant object recognition. Although typically investigated in primates, recent work has shown that rats can be trained in a variety of visual object and shape recognition tasks. These studies did not pinpoint the complexity of the features used by these animals. Many tasks might be solved by using a combination of relatively simple features which tend to be correlated. Alternatively, rats might extract complex features or feature combinations which are nonlinear with respect to those simple features. In the present study, we address this question by starting from a small stimulus set for which one stimulus-response mapping involves a simple linear feature to solve the task while another mapping needs a well-defined nonlinear combination of simpler features related to shape symmetry. We verified computationally that the nonlinear task cannot be trivially solved by a simple V1-model. We show how rats are able to solve the linear feature task but are unable to acquire the nonlinear feature. In contrast, humans are able to use the nonlinear feature and are even faster in uncovering this solution as compared to the linear feature. The implications for the computational capabilities of the rat visual system are discussed.

## Introduction

Starting with the discovery of simple cells by Hubel and Wiesel (1959), decades of neurophysiological research have revealed the coding of a multitude of visual features in the mammalian visual system (Grill-Spector and Malach, 2004) The extraction of these features seems to follow a general principle where simple visual features are coded at the beginning of the visual information processing pathway and tend to be very sensitive to viewing conditions (Rust and Dicarlo, 2010). More complex features appear more upstream and tend to be more robust to different viewing conditions. These complex features are necessary for the visual system to perform tasks such as object recognition (Palmeri and Gauthier, 2004).

However, knowing that a specific feature is detected by the visual system does not necessarily mean that this feature will be used in an object recognition task, even if the object contains that feature. A possible technique to investigate what information is used is the bubbles paradigm, a variant of classification images (Gosselin and Schyns, 2001). The main mechanism behind this technique is to cover the stimulus with a mask so that only parts of it are visible. By allowing the location of the mask to vary from trial to trial and keeping track of the behavioral performance as a function of visible locations, we can determine which object regions are critical in making correct or incorrect identifications.

Previously, we were the first to successfully apply this technique in rats (Vermaercke and Op de Beeck, 2012). After training animals to distinguish between squares and triangles, we demonstrated how rats are capable of applying a flexible recognition template that is invariant to position and size. A more recent study by Alemi-Neissi et al. (2013) extended this approach by training animals on more complex objects. The position, in-plane rotation, azimuth rotation and size were manipulated and for each manipulation the technique was applied to identify the critical image regions for decision making. Their findings revealed that rats are capable of complex object recognition strategies that can include multiple object regions and that are invariant to object view manipulations.

Although the classification image technique is very successful in uncovering which object regions are crucial for object identification or classification, it has two main limitations. First, it only reveals which parts of the image are important, but it does not provide a quantitative indication of the actual features being used. For example, if a subject uses the orientation of a segment for decision making, then the technique will reveal the location at which this segment occurs, but we can only derive that the subject uses the orientation by forming an interpretation of what we see is present at that location. A second limitation is that it only reveals which image regions contribute linearly to performance, which follows directly from how the technique is formally defined. This does not mean that the animal or subject cannot use a feature which contributes in a nonlinear way to performance, it only implies that the technique will not be able to pick up those regions.

In the current study we attempt to work around these limitations by starting from a formal definition of the complexity of the features that are necessary to solve the task. To this end we start with the classical notion of the order of a problem (Minski and Papert, 1969). A problem of order one is a problem in which a single input considered in isolation already contains some information about the desired outcome (Sejnowski et al., 1986). Take the logical AND function as an example. This function has two input units and outputs a one only if each of the input units equals one. If we consider one input unit in isolation there is still some uncertainty about the outcome if it has a value one, but we definitely know that if it has a value zero the output value will also be zero. In that sense the unit contains some information about the actual outcome. In an order two problem, each unit considered in isolation contains no information about the outcome. Taking the prototypical XOR function as an example, it is clear that for any value of one input unit, we still need to know the value of the other input unit in order to get the desired output. The order of a problem is related to the more common notion of linear separability in the theory of neural networks. Here, a classical result is that a perceptron network is able to solve all first order problems but fails to solve the XOR-problem.

If we work with the notion of the order of a problem and apply it to visual stimuli, a first order stimulus set is one for which a single pixel contains some information about the desired outcome. For example, the initial square vs. triangle discrimination in the study of Vermaercke and Op de Beeck (2012) can be considered a first order problem, because pixel intensity levels near the top of the triangle are sufficient to solve the discrimination. Studies of invariant object recognition avoid such simple strategies by transforming the images in various ways so that single pixels are no longer informative. Typical transformations include translations and size changes. Such transformations can be relatively easily compensated for by a visual system by pooling across e.g., positions and size (Riesenhuber and Poggio, 1999). After such normalization the actual decision can again be characterized as a first-order problem.

In contrast, there are problems of a higher order for which features have to be used which are a nonlinear combination of simpler features. An example of a second order stimulus set is one that involves reflection symmetry. Deciding whether a shape has reflection symmetry cannot be done by considering each side of the axis of symmetry in isolation. From this point of view, symmetry detection can be considered as a second-order problem. Note, however, that this is only true for a system which has to work with a sensory coding that is not sensitive to symmetry. If the sensory coding would involve symmetry detectors, then again the actual decision based on the output of such symmetry detectors would become first-order. The symmetry detection itself would require a nonlinear combination of simpler features such as pixel values.

In the present study, we used a stimulus set and tasks which dissociate a simple first-order problem which can be solved using one or more pixel dimensions, from a more complex second-order symmetry-like problem which requires the nonlinear combination of pixel dimensions. We compare rats and human observers in terms of the relative speed by which they learn the two types of problems.

## Materials and Methods

### Stimuli

We carefully constructed a set of four different stimuli. Each stimulus consisted of a central vertical arm. On both the left and the right side, a horizontal arm was positioned, either near the top or the bottom. The stimulus spanned 61.92° horizontally and 69.98° vertically. The width of the central arm measured 17.06°. These measurements are derived on the assumption that rats make their decision when they are approximately 5 cm from the screen. We arrived at this estimate by observing at which distance from the screen rats appear to dwell before making their response. Stimulus brightness is approximately 124 cd/mm^2^ (measured taken with a Minolta CS-100A Chroma Meter).

**Figure 1:**
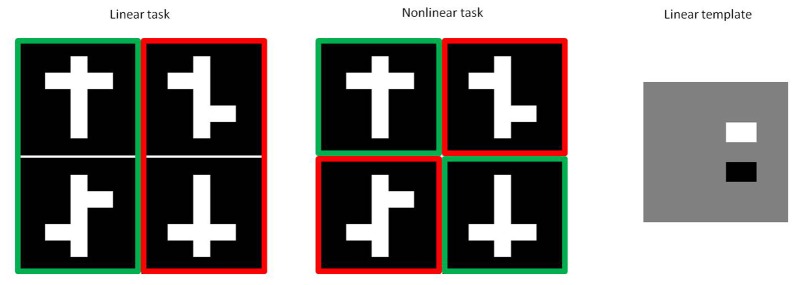
**Experimental stimuli**. Positive stimuli are shown in green, while negative distractor stimuli are shown in red for both the linear (left) and nonlinear task (right). Subtracting the negative stimuli in the linear task from the positive stimuli results in the linear template shown on the right.

Next, we defined two alternative stimulus-response mappings: one mapping can be solved using a simple linear feature (Figure 1, left) and will be called the *linear task*. A possible template that allows to solve this task is presented on the right in Figure 1. The interpretation is that the task could be solved by approaching stimuli with a dark region in the lower right part and/or a bright region in the upper right part.

The second stimulus-response mapping is shown in the middle of Figure 1. Here it is no longer possible to provide a linear template that allows to solve the task. This is the *nonlinear task*. The required decision rule could be verbalized in several ways by referring to dimensions which are not in a linear way related to pixel values, such as alignment of the left and right arms or whether the shapes are symmetrical.

For each pair of stimuli i and j we additionally calculated the pixel-wise distance on the binary coded images (white = 1 and black = 0), using the following formula:

$$Distance_{\text{i,j}}=\frac{\sqrt{\sum {}^{{\text{(}pi-pj\text{)}}^{2}}}}{\sqrt{n}}$$

where the summation is over all corresponding image pixels and the final division provides a normalization for the maximal possible difference in an *n*-dimensional hypercube, where *n* corresponds to the total number of pixels.

For the animals in the linear task we did a further manipulation of the stimulus set in order to retrieve some information on the template that animals were using. Specifically, we systematically added or removed an arm to each positive and negative stimulus in the linear task. This resulted in six unique new positive stimuli and six unique new negative stimuli. From the resulting 36 possible pairwise combinations, we further removed four pairs because the positive and the negative stimulus for that pair were identical. Illustrations of these stimuli are shown in figure × together with the obtained animal performances. These manipulations can be considered as a simplified, low-dimensional version of typical classification image techniques.

### Neural Network Analysis

In order to demonstrate the difference between the two selected stimulus sets, we ran the stimuli through a V1 model and then trained a linear classifier on the output of this model. The model is described in Pinto et al. (2008) and simulation code can be found online. It performs the following steps: a divisive normalization of the input stimulus, convolution with Gabor filters of different orientations and spatial frequencies, output nonlinearities by applying thresholding and response saturation and a final divisive normalization at the output stage.

As a linear classifier we used the basic perceptron learning algorithm. Although more complex algorithms could be used to establish an optimal decision bound, this was not necessary in this case because we only want to demonstrate that one problem is linearly separable. In that case, the perceptron algorithm is guaranteed to converge in finite time.

Finally, the model response was used to calculate an additional measure of stimulus dissimilarity between images i and j using the correlation between filter responses as in the following formula:

Model Distancei,j = 1 − corr (Filter Responsei, Filter Responsej)

### Animal Study

#### Subjects

We used a total of 12 male Long Evans rats in two experiments which differed in how we presented the task stimuli (see “Procedure” Section). Rats were housed in cages of three animals each. Every cage was enriched with a plastic toy item (Bio-Serv, Flemington, NJ, USA). Animals were approximately 3 months old at the start of the experiment. During the experiment all animals received water *ad libitum*. Food intake was restricted to rewards that could be obtained during an experimental session. An additional 15 g of food pellets per animal was administered after each experimental session. For the duration of the experiment, we monitored animal weights and made sure that this did not drop below 85% of their free feeding weight. All experiments were approved by the KU Leuven Animal Ethics Committee (P119/2014). This study did not involve any vulnerable populations.

#### Apparatus

Animals were trained in a touch screen setup (Bussey et al., 2008). This apparatus consists of a trapezoid shaped operant chamber measuring 30.5 cm × 24.1 cm × 8.25 cm. At the small end a reward tray is located. A pellet dispenser allows the delivery of food pellets (45 mg TestDiet sucrose). At the far end is a touch-sensitive display. Access to the screen is restricted by a Perspex plate with two apertures corresponding to the location at which stimuli are shown. The apertures are located x cm above the floor, forcing the animals to rear on their hind legs if they want to make a response. The chamber further includes a tone generator and a house light.

#### Procedure

Animals participated in a single session each day for 5 days per week. The maximum session duration was 60 min. Initially, all animals went through a shaping phase to make them familiar with the operant chamber. This shaping procedure has been well validated and is described more fully in Bussey et al. (2008). Briefly, all animals start with a single session in which food is placed in the reward tray. They are taken out as soon as they have collected all food pellets. All animals then participated in an *Initial touch* session in which a stimulus is shown on either the left or the right part of the screen. When animals touch either of the screens, they receive three reward pellets. If they do not touch the screen within 30 s, the stimulus disappears and they receive only a single reward pellet. After collecting the reward, an inter-trial interval of 20 s starts before the next trial is initiated. In the third session, a stimulus is shown on either the left or the right screen. This time, the stimulus remains on the screen and is only removed if the animal touches the stimulus. Touching the stimulus causes the generation of a tone and the delivery of a single reward pellet. This procedure is repeated until the animal reaches 100 correct trials in a single session. The final part of the shaping procedure is the *Punish incorrect* phase. When animals touch the incorrect position, the house light is illuminated for 5 s, after which an inter-trial interval of 20 s starts. After this timeout, the same trial is presented again and the procedure is repeated until the animal makes a correct response. All trials following an incorrect response (including the final correct trial) are counted as correction trials and disregarded for the analysis.

We then conducted two experiments which differed in when the experimental stimuli were introduced to the animals. In *Experiment A*, animals of the linear and the nonlinear condition both received the same initial pair (first row in Figure 1) in the *initial pair* condition. After behavioral performance reached a criterion performance of more than 80% correct over two consecutive sessions, both groups received the same second pair (second row in Figure 1) in the *second pair* condition. However, for this second pair the target stimuli for the linear condition corresponds to the non-target stimulus in the nonlinear condition. If animals again reached 80% correct, all pairwise combinations were presented in the *mixed pairs* condition. In *Experiment B*, we used the same overall stimulus set for the linear and the nonlinear condition. In contrast with experiment A, all animals started immediately in the mixed pairs condition where both stimulus pairs were shown intermixed.

Six rats participated in Experiment A, three for the linear and three for the nonlinear task, and six rats participated in Experiment B, again three for the linear and three for the nonlinear task.

### Human Study

#### Subjects

Data was collected from 34 university students (age range 18–29 years old; 4 males) of the faculty of psychology who participated in return for course credit. All participants had normal or corrected-to-normal vision. Participants were allocated at random to the linear and the nonlinear task. The experiment was approved by the ethical commission of KU Leuven (G-2015 09 334) and each participant signed an informed consent before the start of the experiment.

#### Apparatus

Experiments took place on a laptop computer running the Matlab (The MathWorks Inc., Natick, MA, USA, 2000) Psychophysics toolbox program (Brainard, 1997). The monitor was a 1600 by 900 LED monitor running at a refresh rate of 60 Hz.

#### Procedure

The stimulus presentation procedure was adapted in order to provide a close match with the quality and resolution of stimulus perception in rats. Target and distractor stimuli were presented to the left and right side of a white fixation cross against a gray background for three frames. Stimuli were then masked using a noise image with 1/f frequency spectrum for x frames. The reasoning behind the eccentric and fast stimulus presentation is that the human visual acuity under these challenging spatial and temporal conditions is not better than the reported visual acuity of rats (Vermaercke and Op de Beeck, 2012). Participants could then use the left or the right arrow key to indicate which position they thought was the correct position. The fixation cross turned green or red if a correct or incorrect response was made. To familiarize the participants with the presentation procedure, we did a shaping session in which a random figure appeared on either the left or the right side of the fixation cross and they had to indicate at which side the stimulus appeared. We kept a running average of the past 20 trials and participants proceeded to the actual experiment if this average exceeded 80%.

At the start of the actual experiment, participants were told that they would see a figure at the left and right side of the fixation cross and that if the correct figure was chosen, the fixation cross would turn green. Their goal was to learn to choose the stimuli for which the fixation cross turned green. So participants had no information regarding the nature of the stimuli. Both participants in the linear and the nonlinear condition received all possible pairwise stimulus combinations intermixed, similar to rats in Experiment B. Stimulus presentation was pseudo-randomized so that every 12 trials, each pair was presented three times. Stimulus location was pseudo-randomized independently from the stimulus pair so that in each block of 12 trials, the correct stimulus for that trial appeared six times to the left side and six times to the right side of the fixation cross. As in the training procedure, we kept a running average of the last 20 trials and training was considered complete as soon as this average exceeded 80%.

### Modeling of Learning Curves

Backward learning curves (Smith and Ell, 2015) for humans were obtained by taking the trial at which criterion performance was reached and including the 19 trials before that trial. For animals we took the session at which the animal reached criterion performance and then included the eight sessions before that session. This number was based on the minimum number of sessions that an animal needed to complete the task in the experiment. This means that each point on the curve is based on the same number of animals/humans but that for a single point, subjects differ in the number of trials they have been in the experiment at that point.

To quantify the shape of these curves we fit a logistic growth curve using a nonlinear least squares optimization algorithm. This model has three different parameters: the asymptotical performance level *(L)*, the learning rate *(k)* and the half-way point between baseline performance and asymptotical performance (*x*_0_). Model fitting was done with the nls2 package in RStudio using the port algorithm (RStudio Team, 2015). To estimate the parameters of the model, initial values need to be given together with constraints on the lower and upper boundary values (see Table 1). The formula for the logistic growth model is:

$$0.55+\frac{0.55L}{1+e^{-k(x-x_{0})}}$$

**Table 1:** **Model fitting parameters**.

	*L*	*k*	*x*_0_
Start	0.5	0.3	10 (5)
Lower	0	1	0
Upper	1	−1	20 (10)

*For x_0_, values differ for humans and rats. Here, values between brackets indicate parameter values used for humans*.

Because visual inspection of the learning curves revealed that the constraints imposed on the parameters would lead to unreasonable results for fitting the nonlinear curve for rats, and because it was clear that the animals did not learn in this task, we fitted a linear curve through the data.

## Results

### Model Analysis

**Figure 2:**
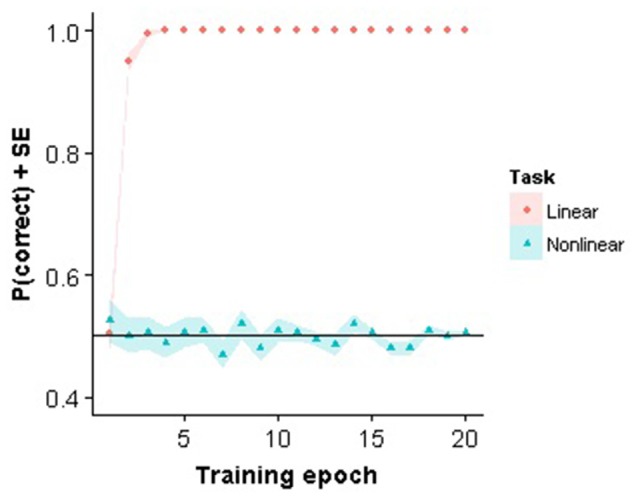
**Perceptron training**. Training performance for the first 20 training epochs for the linear and nonlinear task are shown.

The linear classifier only needed a small number of iterations before the weights were stable when trained on the linear task with the same stimuli as presented to the subjects. In contrast, the linear classifier failed to learn to solve the nonlinear task (Figure 2). Interestingly, in Pinto et al. (2008) it was demonstrated that this V1 model performed reasonably well on different object recognition tasks, even when compared to other more advanced models. Nevertheless, the model fails using our very small stimulus set which confirms that the task is highly non-trivial for a visual system to solve.

Note that using the model implies two assumptions about how information is processed. First, it assumes that cortical decision areas can perform a direct read-out of V1 activity. Experiments have demonstrated that lesions in visual cortex, but not lesions in extrastriate cortex, affect behavioral performance of rats in image and orientation discrimination tasks (Petruno et al., 2013). This seems to suggest that this assumption is valid. A second assumption is that decision areas use a linear readout of V1. At least in monkey IT, the assumption of such a linear readout seems justified (Majaj et al., 2015). Nevertheless, we do not know if the same is true for V1, and if specific task requirements can enforce a different readout mechanism.

**Figure 3:**
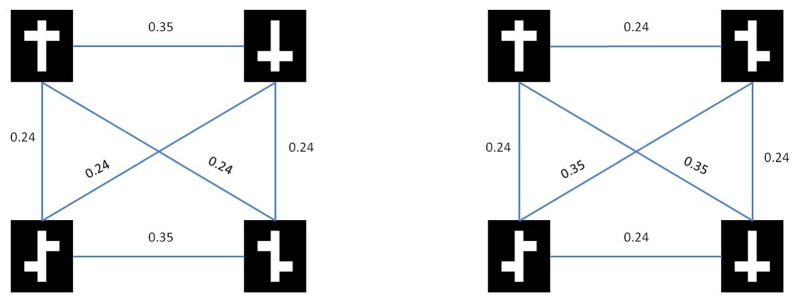
**Stimulus similarities**. Rows represent target (top) and distractor (bottom) stimuli for the nonlinear condition (left) and the linear condition (right).

In Figure 3 we show the results from both the pixel distance metric as well as the model based dissimilarity metric. Note that for the nonlinear task, both metrics show a higher average within-class distance than a between-class. In other words, with respect to low-level stimulus information the two symmetrical stimuli are more dissimilar than a symmetrical vs. an asymmetrical stimulus.

### Learning Curves

**Figure 4:**
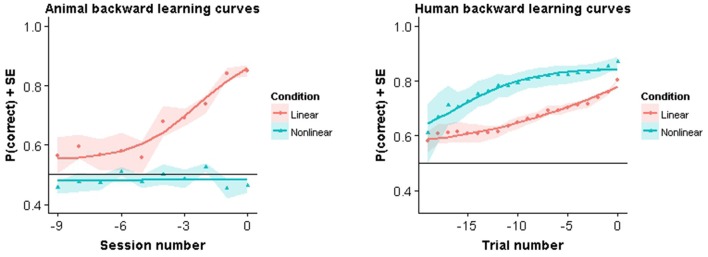
**Backward learning curves**. Solid lines represent fitted models. Symbols represent performance averaged across subjects and color bands represent standard error around the mean.

In Figure 4 we show the backward learning curves for both animals and humans. The animal curves are similar to the modeling results in that all animals in the linear task reach criterion performance, while none of the animals in the nonlinear condition achieved criterion. Training in the linear task lasted on average 20.33 training sessions (SE = 1.96). After all animals in the linear condition had completed the experiment the nonlinear group was still performing at chance level. At that point it was decided to stop training. Nonlinear group animals in Experiment A had been trained for 13.67 (SE = 1.45) sessions in the mixed pair condition and in Experiment B for 36.67 sessions (SE = 0.88) with all pairwise combinations.

Very different results were observed in humans. Not only were they able to acquire the task in both conditions, they also showed a clear advantage in the nonlinear condition. More specifically, in the linear condition, human subjects took on average 46.78 trials (SE = 6.43) to complete the experiment. In contrast, human subjects in the nonlinear condition took on average 24.18 trials (SE = 2.00) trials to complete the experiment. This difference was found to be significant using the Welch two sample *t*-test (*t*_(15.52)_ = 3.53, *p* = 0.004). Thus, in humans, learning was faster for the nonlinear task.

This observation is also reflected in the estimated parameters for the backward learning curves (see Table 2). Specifically, a higher learning rate is observed together with a lower estimation of the midpoint between baseline and asymptotical performance. Note however that for the different learning curves we are missing data points at baseline performance and asymptotical performance. Therefore the absolute values of these parameters are probably less useful for interpretation.

**Table 2:** **Resulting parameter values obtained by fitting the logistic growth model to the backward learning curves**.

	*L*	*k*	*x_0_*	SSE
Animals	0.68	0.68	6.65	0.006
Humans-linear	0.89	0.12	20	0.002
Humans-nonlinear	0.54	0.27	2.68	0.003

### Interspecies Performance Comparisons

**Figure 5:**
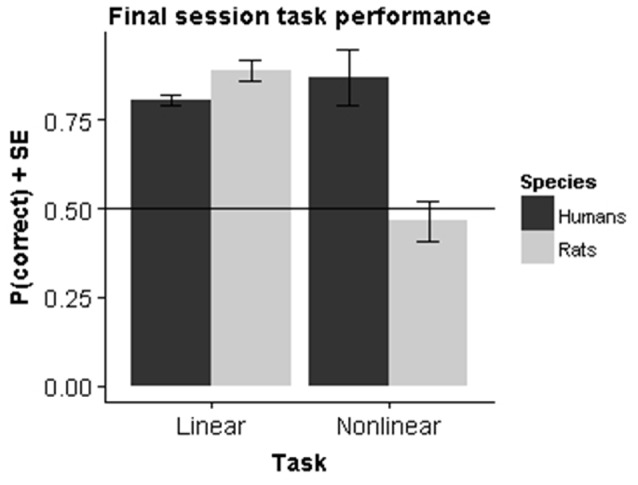
**Species comparison**. Bars represent the mean performance across subjects during their final session in the experiment.

To assess performance differences we performed a two-way analysis of variance (ANOVA) with factor Species (rats vs. humans) and Task (linear vs. nonlinear) on the performance in the final session. The results (Figure 5) showed a significant main effect for both Species (*F*_(1,39)_ = 77.20, *p* < 0.001) and Task (*F*_(1,39)_ = 18.67, *p* < 0.001) as well as a significant interaction effect (*F*_(1,39)_ = 176.65, *p* < 0.001).

The main effect of species is not very relevant, as the final performance is very sensitive to the exact testing procedure and the criterion used to decide to stop testing. In particular, it is obvious that humans learn much faster overall, completing training in a few tens of trials. The most important effect is the significant cross-over interaction. Follow-up tests show that within the linear task rats had a significantly higher final performance compared to humans (*F*_(1,19)_ = 82.46, *p* < 0.001), whereas the opposite was true for the nonlinear task (*F*_(1,19)_ = 133.6, *p* < 0.001). The superior performance for rats in the linear task can be explained by the criterion that we set for task learning. In humans, the criterion is sufficient to establish that humans perform above chance. However, the learning curves together with the fit from the logistic growth model indicate that they have not yet reached asymptotical performance. If we had used a longer number of trials, it is likely that the difference would disappear. The superior performance in the nonlinear task is more important, because it is clear that animals don’t learn the task at all. Within species, there was also a very significant effect of task in rats (*F*_(1,10)_ = 273.1, *p* < 0.001), which contrasts with a significant effect in the other direction for humans (*F*_(1,29)_ = 10.12, *p* < 0.001).

We additionally ran a two-way ANOVA with factor Species and Task on reaction times from the final session. This revealed a significant main effect of Species (*F*_(1,39)_ = 197.68, *p* < 0.001) but no main effect of Task (*F*_(1,39)_ = 1.124, *p* = 0.30) or interaction between Species and Task (*F*_(1,39)_ = 0.38, *p* = 0.54). Humans took on average 0.47 (SE = 0.005) seconds to respond, while animals took on average 2.54 (SE = 0.065) seconds to respond. This effect is related to the differences in experimental procedures as rats have to move a certain physical distance before they can make a response.

### Animal Behavior Strategy in the Nonlinear Task

**Figure 6:**
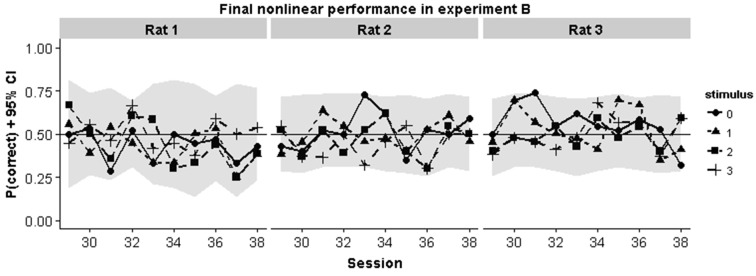
**Animal performance during the final 10 training sessions in the nonlinear condition of Experiment B**. Individual performances for each pair are shown. They gray region indicates the smallest binomial confidence interval that could be obtained given the number of trials for each pair in that session.

To better understand why animals were failing in the nonlinear task we inspected the performance curves on each pair in the final 10 sessions in Experiment B. Chance performance could in principle arise because animals perform at chance level for all stimulus pairs. Alternatively, they might apply a linear rule which would lead to above chance performance for some stimuli and below chance performance for other stimuli. In each case, mean session performance would be around chance level. The curves in Figure 6 show that the first possibility is the case: animals consistently perform around chance for all stimulus pairs.

**Figure 7:**
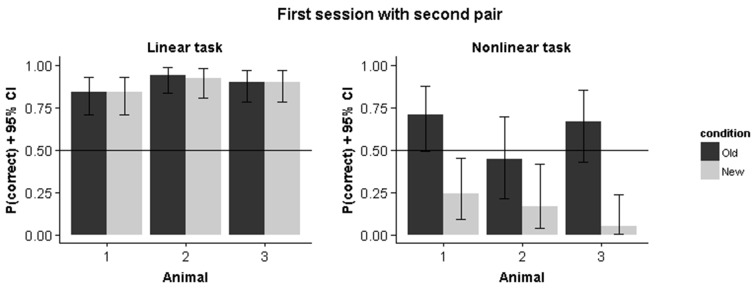
**Performance on the first pair and the second pair in the first session that the second pair was introduced**. Error bars represents 95% binomial confidence intervals.

Results from Experiment A, in which we started training with a single stimulus pair in each condition, indicates that the rats in the nonlinear condition initially pick up a linear decision rule. Introducing the second pair leads to different performance predictions in the linear vs. the nonlinear condition. For the former, the new target is consistent with the linear rule obtained on the first pair and we expect the same performance on the old vs. the new pair. For the nonlinear condition, this same decision rule would lead to a preference for the non-target stimulus. Figure 7 shows how animals immediately perform above chance for the new pair while performance is below chance for this pair in the nonlinear condition. We calculated the correlation between old and new pair performance for the remaining sessions in the nonlinear condition. This revealed a negative correlation between performance on the old pair and the new pair (*r* = −0.44, *p* = 0.002), which further indicates how the strategy used to solve the old stimulus pair is detrimental for solving the new stimulus pair.

### Animal Behavioral Strategy in the Linear Task

**Figure 8:**
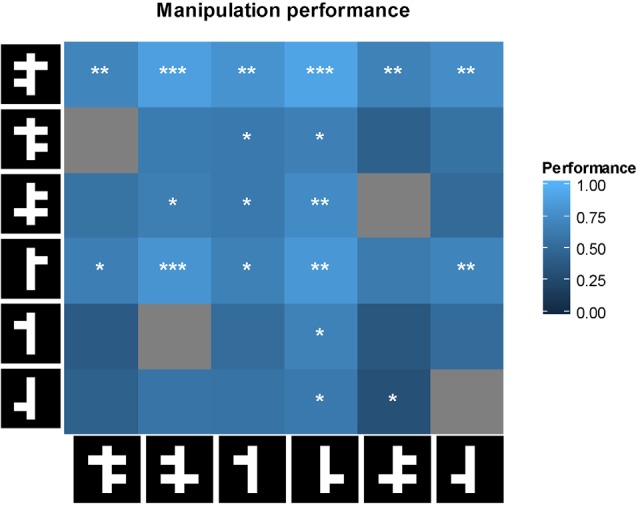
**First row: performance of the animal on each of the manipulated stimulus pairs**. For each cell, performance was tested with a two-tailed *t*-test against chance performance. Significance values are indicated as follows: *p < 0.05, **p < 0.01, ***p < 0.001. Gray cells are not shown because they involve two identical stimuli. On row 5, columns 2 and 3, an identical stimulus pair was shown and a non-identical pair was not shown because of a software error.

Performance in the linear task can be predicted by looking either at the top or bottom part of the stimulus, or both. However, each strategy predicts a different response to the manipulated stimuli with arms added or removed. These predictions can be tested by computing correlations across the cells in the matrix in Figure 8 between animal performance and the predictions of a model based upon a particular strategy. For example, a model based upon a strategy which only takes the bottom part of the stimulus into account, predicts chance performance if the two stimuli in a pair do not differ in their bottom parts. The results for the model correlations were as follows: *r* = 0.66 (*p* < 0.001) for the full model, *r* = 0.08 (*p* = 0.65) for the lower visual field model, and *r* = 0.73 (*p* < 0.001) for the upper visual field model. Thus, the six animals seem to use the top of the stimuli in the linear task.

## Discussion

Using an object discrimination task, we probed the capacity of rats to use qualitatively different types of stimulus features. In the linear condition, a simple local cue was sufficient to solve the task. In the nonlinear condition, solving the task required a nonlinear combination of two parts of the visual stimulus. A simulation with a V1 model highlighted the differences between the two tasks. Both animal and human subjects were able to learn the linear task. The nonlinear task was never picked up by the animals. In contrast, humans acquired the nonlinear task and even learned this task much faster than the linear task.

Why did the animals fail to learn the nonlinear task? There are several alternative explanations. A first hypothesis is that rats do not have a cortical representation that is related to the symmetry of the stimulus. Such a signal is interesting because it essentially turns the categorization problem in a linear problem that can be solved by a simple readout mechanism. However, constructing a representation that relates to symmetry is no trivial task. Our own modeling results indicated that processing mechanisms in V1 are insufficient. Likewise, models that have been designed to calculate an explicit symmetry signal contain make use of computations that are not known to exist in primary visual areas (Poirier and Wilson, 2009). In an fMRI study, Sasaki et al. (2005) were unable to find symmetry related activity in early visual areas. Only starting in V3, and further in V4, V7 and LO did observe modulations related to the symmetry of the visual stimulus. So although the nonlinear task could be solved with apparent ease by humans using the concept of symmetry, the underlying mechanisms that allow them to do so are quite complex and could be absent in rats.

The latter would be a surprising conclusion, given that the ability to use, or even prefer, symmetry seems to be ubiquitous in the animal kingdom. For example, Delius and Habers (1978) trained pigeons on a set of symmetrical vs. asymmetrical images. Although their training set might not be strictly nonlinear as in our task, a further generalization test indicated that animals were using symmetry as a concept for solving the categorization task. Similar capabilities are found in a wide range of species, including other birds (Swaddle and Pruett-Jones, 2001; Mascalzoni et al., 2012), fish (Schluessel et al., 2014) and insects (Giurfa et al., 1996; Rodríguez et al., 2004). The relation between the ability to detect symmetry and symmetry preference is less clear, as it has been found to be absent in some species (Schluessel et al., 2014), and even a preference for the asymmetrical shapes has been reported (Delius and Nowak, 1982). Interestingly, symmetry detection has been reported in animals that have an even lower spatial acuity than rats (Macuda et al., 2001), which rules out that a possible absence of symmetry is related to spatial acuity.

It is interesting to compare two ecological factors that are related to symmetry perception. One important factor is that symmetrical features are often linked to attractiveness or preferential mate selection. Both humans and monkeys seem to prefer symmetrical faces over asymmetrical faces (Mealey et al., 1999; Waitt and Little, 2006), and similar phenomena have been observed in birds (Møller, 1992; Swaddle and Cuthill, 1994) and fish (Merry and Morris, 2001; Mazzi et al., 2003). In that respect it is interesting to note that as far as male preference for female rats is concerned, one study has found no evidence that male rats prefer any particular female (Chu and Ågmo, 2015). Using a more controlled setting Edwards et al. (1990) found that male rats did prefer a receptive female over a non-receptive female. However removing of the olfactory bulb eliminated this preference. Together these studies indicate that symmetry is not an important factor in rat mate selection, which seems more determined by olfactory cues.

Another important ecological factor relates to foraging behavior. This has been demonstrated in bees, which prefer symmetrical flowers over nonsymmetrical ones (Wignall et al., 2006). In rats, again, preference for food seems to be determined largely by odor. Experiments have demonstrated that they are able to detect the smell of food on another rat and will subsequently prefer food that has been eaten by another rat (Galef and Wigmore, 1983; Posadas-Andrews and Roper, 1983). In sum, two major ecological factors that could potentially drive the development of symmetry detection mechanisms seem to be solved by olfactory cues in rodents.

However, animals do not necessarily need to be able to use the concept of symmetry in order to solve the task. In the “Introduction” Section we set out to describe the symmetry task as a task that is nonlinear with respect to the low-level visual features that make up the task. The question then becomes to what extent animals are able to solve nonlinear visual classification problems. This ability has been investigated explicitly in both human and nonhuman primates. In Smith et al. (2011) colored disks were used, for which a green-yellow and blue-red disk belonged to category A and a blue-yellow and green-red disk belonged to category B. Thus, whether a stimulus belongs to category A or B depends crucially on the particular combination of colors and not on the individual colors that make up the circle. For these stimuli humans do not have a concept available that could make the classification problem easy to solve. Indeed, results indicated that learning this category problem was difficult. Nevertheless, humans eventually learned to solve the problems. Monkeys could also learn the classification problem but at reduced performance levels, leading the authors to conclude that these animals are biased towards linear separable categories. A similar result has been found in pigeons (Cook and Smith, 2006) when presented with categories of colored circles with exception-items. These items are stimuli that share more features with the opposite category than with the one which they actually belong, which essentially creates a nonlinear category problem. Pigeons showed difficulties with acquiring the correct category structure but learned to do so eventually. Together, these studies indicate that although a linear separability bias can exist, it is not something that cannot be overcome. Thus, our results could provide an indication that rats bring a linear separability constraint to visual classification problems that is too strong to overcome.

Alternatively, failure to learn the task could be related to the strategy that animals employ to reach a solution. Several studies have demonstrated that in visual discrimination tasks, rats seem to default to a similarity based strategy. For example, when comparing a rule-based categorization task with an information integration categorization task, Vermaercke et al. (2014) showed that animals solve these task using a similarity based strategy. In that experiment, this lead to a better performance for animals when compared with humans doing a similar task. So a similarity based strategy can be a very useful strategy in some circumstances. However, it is not useful in the present context when the similarity is computed using simple low-level features. Indeed, using both pixel and model dissimilariry indices we showed that the similarity between target and distractor is higher for the nonlinear task than for the linear task. Thus, a strategy that tries to exploit this similarity would be expected to have more difficulties with the nonlinear task.

Our findings might seem to stand in contrast with recent studies that show how rats are capable of invariant object recognition (Zoccolan et al., 2009; Zoccolan, 2015). How is it that they can distinguish between two objects despite large variations in viewing conditions, yet fail to discriminate using only a small stimulus set such as the one we used in our experiment? This apparent contradiction can be resolved if we take into account that in these studies, training typically begins with a prototypical stimulus object which the animal first learns to discriminate. Training then proceeds by including minor variations in object views. By its very nature, different object views will contain at least some overlap with each other which the visual system can exploit to learn which object view belong to which object. This same strategy of exploiting object similarity is actually detrimental in our nonlinear task.

Finally, it should also be mentioned that rats failed to employ two other strategies that could in principle be used to solve the task (Smith et al., 2011). A first potential strategy is the rule-based strategy, which can be defined as looking for an explicit rule. This is an important strategy for humans which recruits the prefrontal cortex (Ashby and Maddox, 2005). However, already in tasks that can be solved by a simple unidimensional rule (and thus benefit from such a strategy), it was suggested that rats do not show a preference for this strategy (Vermaercke et al., 2014). It is thus very unlikely that they will resort to a rule-based strategy if the task becomes even more complex. Furthermore, a rule has to refer to concrete stimulus features. If this is a stimulus feature such as “symmetry” which the rats might not extract, then the failure to find the rule is actually due to a failure to extract the features on which the rule is defined. As a second missed strategy, rats could store specific exemplar views and map these exemplars to correct responses. Given the limited size of the stimulus set, it is unclear why rats fail to employ this strategy. One explanation could be the conflict with the aforementioned strategy based upon low-level similarity.

Our experiment is not the first in which it is demonstrated that rats fail in some basic visual tasks. For example, in Minini and Jeffery (2006) it was demonstrated that rats showed difficulties using a configural cue such as aspect ratio for doing stimulus discrimination. Although an explicit comparison with humans was not included, it is safe to assume that using concepts such as squares and rectangles this task could easily be solved by humans. Also, Meier and Reinagel (2013) showed that detection of a grating is impaired in rats if presented with collinear features, while in humans this typically leads to better performance. Together, our findings show that despite several global similarities between the simple rat visual system and more complex human visual system, there are edge cases which illustrate that differences do exist. An interesting question for further research is to find out how these differences in behavioral performances can be linked to differences in cortical architecture.

## Author Contributions

HPOB conceived the study. CB designed the stimuli and performed the data analysis. CB and HPOB wrote the article.

## Funding

This research was supported by Fonds Wetenschappelijk Onderzoek (FWO) grants G.0819.11 and G.0A39.13, the Research Council Leuven (grant GOA/12/008), and Hercules Foundation Award AKUL/13/06.

## Conflict of Interest Statement

The authors declare that the research was conducted in the absence of any commercial or financial relationships that could be construed as a potential conflict of interest.

## References

[B1] Alemi-Neissi A. Rosselli F. B. Zoccolan D. (2013). Multifeatural shape processing in rats engaged in invariant visual object recognition. J. Neurosci. 33, 5939–5956. 10.1523/JNEUROSCI.3629-12.2013 23554476 PMC6618908

[B2] Ashby F. G. Maddox W. T. (2005). Human category learning. Annu. Rev. Psychol. 56, 149–178. 10.1146/annurev.psych.56.091103.070217 15709932

[B3] Brainard D. H. (1997). The psychophysics toolbox. Spat. Vis. 10, 433–436. 10.1163/156856897x00357 9176952

[B4] Bussey T. J. Padain T. L. Skillings E. A. Winters B. D. Morton A. J. Saksida L. M. (2008). The touchscreen cognitive testing method for rodents: how to get the best out of your rat. Learn. Mem. 15, 516–523. 10.1101/lm.987808 18612068 PMC2505319

[B5] Chu X. Ågmo A. (2015). Sociosexual behaviors of male rats (Rattus norvegicus) in a seminatural environment. J. Comp. Psychol. 129, 132–144. 10.1037/a0038722 25621492

[B6] Cook R. G. Smith J. D. (2006). Stages of abstraction and exemplar memorization in pigeon category learning. Psychol. Sci. 17, 1059–1067. 10.1111/j.1467-9280.2006.01833.x 17201788

[B7] Delius J. D. Habers G. (1978). Symmetry: can pigeons conceptualize it? Behav. Biol. 22, 336–342. 10.1016/s0091-6773(78)92411-2 626628

[B8] Delius J. D. Nowak B. (1982). Visual symmetry recognition by pigeons. Psychol. Res. 44, 199–212. 10.1007/bf00308420 7156265

[B9] Edwards D. A. Griffis K. T. Tardivel C. (1990). Olfactory bulb removal: effects on sexual behavior and partner-preference in male rats. Physiol. Behav. 48, 447–450. 10.1016/0031-9384(90)90342-2 2267253

[B10] Galef B. G. Jr. Wigmore S. W. (1983). Transfer of information concerning distant foods: a laboratory investigation of the information-centre hypothesis. Anim. Behav. 31, 748–758. 10.1016/s0003-3472(83)80232-2

[B11] Giurfa M. Eichmann B. Menzel R. (1996). Symmetry perception in an insect. Nature 382, 458–461. 10.1038/382458a0 18610516

[B12] Gosselin F. Schyns P. G. (2001). Bubbles: a technique to reveal the use of information in recognition tasks. Vision Res. 41, 2261–2271. 10.1016/s0042-6989(01)00097-9 11448718

[B13] Grill-Spector K. Malach R. (2004). The human visual cortex. Annu. Rev. Neurosci. 27, 649–677. 10.1146/annurev.neuro.27.070203.144220 15217346

[B14] Hubel D. H. Wiesel T. N. (1959). Receptive fields of single neurones in the cat’s striate cortex. J. Physiol. 148, 574–591. 10.1113/jphysiol.1959.sp006308 14403679 PMC1363130

[B15] Macuda T. Gegear R. Laverty T. Timney B. (2001). Behavioural assessment of visual acuity in bumblebees *(Bombus impatiens)*. J. Exp. Biol. 204, 559–564. 11171306 10.1242/jeb.204.3.559

[B16] Majaj N. J. Hong H. Solomon E. A. DiCarlo J. J. (2015). Simple learned weighted sums of inferior temporal neuronal firing rates accurately predict human core object recognition performance. J. Neurosci. 35, 13402–13418. 10.1523/JNEUROSCI.5181-14.2015 26424887 PMC4588611

[B17] Mascalzoni E. Osorio D. Regolin L. Vallortigara G. (2012). Symmetry perception by poultry chicks and its implications for three-dimensional object recognition. Proc. Biol. Sci. 279, 841–846. 10.1098/rspb.2011.1486 21920978 PMC3259931

[B18] Mazzi D. Künzler R. Bakker T. C. M. (2003). Female preference for symmetry in computer-animated three-spined sticklebacks, *Gasterosteus aculeatus*. Behav. Ecol. Sociobiol. 54, 156–161. 10.1007/s00265-003-0609-0

[B19] Mealey L. Bridgstock R. Townsend G. C. (1999). Symmetry and perceived facial attractiveness: a monozygotic co-twin comparison. J. Pers. Soc. Psychol. 76, 151–158. 10.1037/0022-3514.76.1.151 9972560

[B20] Meier P. M. Reinagel P. (2013). Rats and humans differ in processing collinear visual features. Front. Neural Circuits 7:197. 10.3389/fncir.2013.00197 24379758 PMC3862114

[B21] Merry J. W. Morris M. R. (2001). Preference for symmetry in swordtail fish. Anim. Behav. 61, 477–479. 10.1006/anbe.2000.1589

[B22] Minini L. Jeffery K. J. (2006). Do rats use shape to solve “shape discriminations”? Learn. Mem. 13, 287–297. 10.1101/lm.84406 16705141 PMC1475809

[B23] Minski M. L. Papert S. A. (1969). Perceptrons: An Introduction to Computational Geometry. Cambridge, MA: MIT Press.

[B24] Møller A. P. (1992). Female swallow preference for symmetrical male. Nature 357, 238–240. 10.1038/357238a0 1589021

[B25] Palmeri T. J. Gauthier I. (2004). Visual object understanding. Nat. Rev. Neurosci. 5, 291–303. 10.1038/nrn1364 15034554

[B26] Petruno S. K. Clark R. E. Reinagel P. (2013). Evidence that primary visual cortex is required for image, orientation and motion discrimination by rats. PLoS One 8:e56543. 10.1371/journal.pone.0056543 23441202 PMC3575509

[B27] Pinto N. Cox D. D. DiCarlo J. J. (2008). Why is real-world visual object recognition hard? PLoS Comput. Biol. 4:e27. 10.1371/journal.pcbi.0040027 18225950 PMC2211529

[B28] Poirier F. J. Wilson H. R. (2009). A biologically plausible model of human shape symmetry perception. J. Vis. 10:9. 10.1167/10.1.9 20143902

[B29] Posadas-Andrews A. Roper T. J. (1983). Social transmission of food-preferences in adult rats. Anim. Behav. 31, 265–271. 10.1016/s0003-3472(83)80196-1

[B30] Riesenhuber M. Poggio T. (1999). Hierarchical models of object recognition in cortex. Nat. Neurosci. 2, 1019–1025. 10.1038/14819 10526343

[B31] Rodríguez I. Gumbert A. Hempel de Ibarra N. Kunze J. Giurfa M. (2004). Symmetry is in the eye of the “beeholder”: innate preference for bilateral symmetry in flower-naïve bumblebees. Naturwissenschaften 91, 374–377. 10.1007/s00114-004-0537-5 15278213

[B300] RStudio Team (2015). RStudio: Integrated Development for R. Boston, MA: RStudio, Inc., Available online at: http://www.rstudio.com

[B32] Rust N. C. Dicarlo J. J. (2010). Selectivity and tolerance (“invariance”) both increase as visual information propagates from cortical area V4 to IT. J. Neurosci. 30, 12978–12995. 10.1523/JNEUROSCI.0179-10.2010 20881116 PMC2975390

[B33] Sasaki Y. Vanduffel W. Knutsen T. Tyler C. Tootell R. (2005). Symmetry activates extrastriate visual cortex in human and nonhuman primates. Proc. Natl. Acad. Sci. U S A 102, 3159–3163. 10.1073/pnas.0500319102 15710884 PMC549500

[B34] Schluessel V. Beil O. Weber T. Bleckmann H. (2014). Symmetry perception in bamboo sharks *(Chiloscyllium griseum)* and Malawi cichlids (*Pseudotropheus* sp.). Anim. Cogn. 17, 1187–1205. 10.1007/s10071-014-0751-2 24794621

[B35] Sejnowski T. J. Kienker P. K. Hinton G. E. (1986). Learning symmetry groups with hidden units: beyond the perceptron. Physica D 22, 260–275. 10.1016/0167-2789(86)90245-9

[B37] Smith J. D. Coutinho M. V. Couchman J. J. (2011). The learning of exclusive-or categories by monkeys (Macaca mulatta) and humans (Homo sapiens). J. Exp. Psychol. Anim. Behav. Process. 37, 20–29. 10.1037/a0019497 20718556 PMC3531230

[B36] Smith J. D. Ell S. W. (2015). One giant leap for categorizers: one small step for categorization theory. PLoS One 10:e0137334. 10.1371/journal.pone.0137334 26332587 PMC4558046

[B38] Swaddle J. P. Cuthill I. C. (1994). Female zebra finches prefer males with symmetric chest plumage. Proc. R. Soc. Lond. B Biol. Sci. 258, 267–271. 10.1098/rspb.1994.0172

[B39] Swaddle J. P. Pruett-Jones S. (2001). Starlings can categorize symmetry differences in dot displays. Am. Nat. 158, 300–307. 10.1086/321323 18707326

[B41] Vermaercke B. Cop E. Willems S. D’Hooge R. Op de Beeck H. P. (2014). More complex brains are not always better: rats outperform humans in implicit category-based generalization by implementing a similarity-based strategy. Psychon. Bull. Rev. 21, 1080–1086. 10.3758/s13423-013-0579-9 24408657

[B40] Vermaercke B. Op de Beeck H. P. (2012). A multivariate approach reveals the behavioral templates underlying visual discrimination in rats. Curr. Biol. 22, 50–55. 10.1016/j.cub.2011.11.041 22209530

[B42] Waitt C. Little A. C. (2006). Preferences for symmetry in conspecific facial shape among *Macaca mulatta*. Int. J. Primatol. 27, 133–145. 10.1007/s10764-005-9015-y

[B43] Wignall A. E. Heiling A. M. Cheng K. Herberstein M. E. (2006). Flower symmetry preferences in honeybees and their crab spider predators. Ethology 112, 510–518. 10.1111/j.1439-0310.2006.01199.x

[B44] Zoccolan D. (2015). Invariant visual object recognition and shape processing in rats. Behav. Brain Res. 285, 10–33. 10.1016/j.bbr.2014.12.053 25561421 PMC4383365

[B45] Zoccolan D. Oertelt N. DiCarlo J. J. Cox D. D. (2009). A rodent model for the study of invariant visual object recognition. Proc. Natl. Acad. Sci. U S A 106, 8748–8753. 10.1073/pnas.0811583106 19429704 PMC2679579

